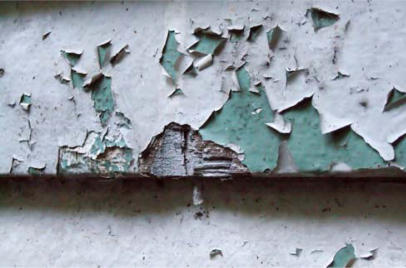# Lead: Dangerous Wait

**DOI:** 10.1289/ehp.115-a298a

**Published:** 2007-06

**Authors:** Tina Adler

You think waiting for the cable guy is bad? Some families must wait more than a year for a lead abatement team to remove or contain the lead that is poisoning their children, reports a group led by Kristina M. Zierold of the Wake Forest University School of Medicine.

The team examined housing data collected between 1996 and 1999 from 382 Wisconsin children with first-time blood lead levels between 20 and 40 μg/dL. Getting rid of the lead from these children’s homes took a median of 465 days, the team writes in the February 2007 issue of the *American Journal of Public Health*. For 45% of the children, abatement took more than 18 months, whereas for 18% of the children, cleanup occurred within 6 months.

Wisconsin, with its strong public health system, is probably as fast if not faster than other states at performing lead abatement, says Zierold. But little research has documented abatement times, so it’s hard to say how fast or slow any state is. If a child has a blood lead level of 20 μg/dL or higher, an environmental intervention should begin within 10 days, according to the CDC. Zierold says local or state health officials are usually prompt about writing abatement orders, but landlords don’t always have the money to do the cleanup.

She and her colleagues found clear racial disparities in the rate of lead abatement. Homes where white children lived were almost twice as likely as the homes of black children to be cleaned up within 6 months (almost 70% of the children in the study were black). Although the authors did not ask participants about socioeconomic status, they note that most black children in Wisconsin live in lower-income urban communities, and that families with lower income tend to reside in rental housing.

“Are these results a surprise? Given the lack of resources we put toward the problem, no,” says Bruce P. Lanphear, director of the Cincinnati Children’s Environmental Health Center. “The question these findings raise is, why do we wait until children are poisoned in the first place? Why don’t we try to identify [contaminated] housing units before the kids are poisoned?” Nationally, 38 million homes contain lead-based paint, and lead poses a risk in 35% of all low-income housing, according to the CDC. Moreover, studies are now showing that blood lead levels as low as 2–3 μg/dL harm children’s physical and mental development; in the past, levels below 10 μg/dL were considered acceptable.

Abatement times have improved since the study began. The authors write that by 1999 lead-safety improvements were completed within 6 months in 31% of homes. According to Zierold, since the study began Wisconsin has made a stronger effort to investigate the homes of children with elevated blood lead within two weeks. The Wisconsin Department of Health and Family Services also launched a registry of single-family homes, apartments, and daycare facilities that meet lead-free or lead-safe property standards. This is particularly important as the families of poor children tend to move frequently, and the registry increases their chances of finding lead-free housing.

## Figures and Tables

**Figure:**